# Neonatal outcomes and immune reprogramming induced by prenatal opioid and hepatitis C exposure

**DOI:** 10.3389/fimmu.2026.1794914

**Published:** 2026-10-13

**Authors:** Brianna M. Doratt, Heather E. True, Sheridan B. Wagner, Delphine C. Malherbe, Cynthia Cockerham, John O’Brien, Ilhem Messaoudi

**Affiliations:** 1 Department of Microbiology, Immunology, and Molecular Genetics, College of Medicine, University of Kentucky, Lexington, KY, United States; 2 Department of Obstetrics & Gynecology, College of Medicine, University of Kentucky, Lexington, KY, United States

**Keywords:** hepatitis C (HCV), maternal opioid use disorder, neonatal immunity, pregnancy, transcriptomics

## Abstract

**Introduction:**

Prenatal opioid exposure (POE) is an escalating public health concern. However, its impact on neonatal immune development remains poorly defined. This study examined how POE, with or without maternal hepatitis C (hepatitis C virus, HCV) infection, shapes neonatal immunity.

**Methods:**

Umbilical cord blood was collected from pregnancies included uncomplicated term controls (n=38) and deliveries in patients with severe opioid use disorder on medication-assisted treatment, with (n=41) or without HCV (n=34) infection. Flow cytometry was used to characterize cell phenotype and functional response to stimulation. Single-cell RNA sequencing was used to investigate transcriptomics changes.

**Results:**

POE was associated with smaller birth size, higher rates of congenital anomalies, and longer intensive care admissions. Immune profiling showed a proinflammatory milieu, reduced circulating levels of neuroprotective factors, expanded memory/effector T and B cells, blunted T-cell responses, and markedly reduced natural killer (NK) cell frequency and cytotoxicity, especially in POE HCV+ newborns. Monocytes exhibited inflammatory reprogramming with impaired antiviral and tissue repair pathways and disrupted lymphoid–innate immune cell communication.

**Discussion:**

These findings suggest that POE-driven immune disequilibrium may underlie adverse early-life outcomes and highlight monitoring and intervention targets.

## Introduction

The rates of opioid use disorder (OUD) among pregnant women are alarmingly high, with approximately 325 incidences per 10,000 live births (1). The rates of neonatal opioid withdrawal syndrome (NOWS) are also increasing, with approximately 60 newborns diagnosed daily in the United States (2, 3). In addition, newborns with prenatal opioid exposure (POE) are at a higher risk of preterm birth, low birth weight, and still birth (3–5). POE is also associated with long-term cognitive and behavioral deficits (3, 5, 6) and an increased risk of immune-related conditions including infections, asthma, and eczema (7). However, the impact of POE on neonatal immunity remains poorly understood.

Studies using rodent models have reported that methadone-exposed rat pups *in utero* exhibited increased baseline production of pro-inflammatory chemokines and cytokines and sustained hyperreactivity and exaggerated cytokine production following lipopolysaccharide (LPS) stimulation (8, 9). Furthermore, prenatal exposure to methadone or buprenorphine impaired neurodevelopment and increased neuroinflammation in rat pups (10). While rodent models offer valuable insights into the mechanisms of neonatal development, significant differences in gestational length, neonatal maturity, and the placental structure limit their translation to human pregnancy (11, 12).

In contrast, studies of peripheral immunity in human neonates with POE are limited. A recent study has shown that neonates with POE have lower levels of cord blood neutrophils and monocytes compared with unexposed controls (13). Moreover, POE has been associated with decreased serum concentrations of inflammatory cytokines in human neonates (13). Furthermore, human umbilical cord blood mononuclear cells (UCBMCs) show reduced IL-2 production by CD4^+^ T cells at baseline and diminished monocyte bacterial killing activity, indicating functional immune senescence (13). These observations are closely aligned with epidemiological studies that reported higher risk of hospitalization due to infection in children with POE (7) and differ significantly from data reported in the rodent studies mentioned above. The discrepancies between findings in rodent models (pro-inflammatory priming) and human neonates (immune suppression) likely reflect critical differences in fetal development and the timing of analysis as rodent studies assessed offspring postnatally (7–60 days post birth), not at delivery. More importantly, these differences emphasize the need for additional in-depth clinical studies.

To address this significant gap in knowledge, we investigated the early neonatal immune environment using umbilical cord blood (UCB) collected at the time of delivery from newborns born to healthy participants (control) and those from pregnant participants with OUD (POE). UCB plasma was assayed for immune mediator levels, while UCBMCs were used to uncover the phenotypic, functional, and transcriptional (single-cell RNA sequencing, scRNA-seq) immune landscape. We next inferred the intercellular communication networks from our scRNA-seq dataset using CellChat (14). Finally, given the high prevalence of hepatitis C (hepatitis C virus, HCV) infection in this cohort, we stratified our findings by maternal HCV status.

## Materials and methods

### Sample collection and processing

This study was approved by the Institutional Ethics Review Board of the University of Kentucky, Lexington, KY (IRB no. 43698—Comprehensive Perinatal Substance Abuse Treatment). All subjects provided signed consent before enrollment in the study. Healthy-term controls were concurrently enrolled as part of a separate study evaluating biomarkers of preterm labor (IRB no. 73519—Prospective Evaluation of Pathways for Preterm Birth: The PEPP Study). All pregnant participants in this study were recruited from the University of Kentucky. Recruitment took place from May 2022 to March 2025. The inclusion criteria were pregnant women aged 18–41 years, between 6 and 36 weeks’ gestation, and a diagnosis of OUD. Plasma and UCBMCs were isolated from UCB samples collected at the time of delivery (>37 weeks gestational age) as previously described (15, 16). Briefly, blood was collected in EDTA vacutainer tubes, stored at 4°C, and processed within 24 h. Cells and plasma were isolated after whole-blood centrifugation over LymphoPrep, Stemcell Technologies, Vancouver, Canada. Plasma was stored at −80°C, and UCBMCs were cryopreserved using 10% dimethyl sulfoxide (DMSO)/fetal bovine serum (FBS) until analysis. The characteristics of the cohort are shown in **Supplementary Table S1**.

### Luminex assay

UCB plasma was collected for Luminex assay according to the manufacturer’s instructions for the following Milliplex MAP human kits, MilliporeSigma, Darmstadt, Germany: Cytokine/Chemokine/Growth Factor Panel A (HCYTA-60K-PX38), Neurological Disorders Magnetic Bead Panel 3 [HND3MAG-39K-02; osteopontin (OPN) and SOD1], and Neuropeptide Magnetic Bead Panel (HNPMAG-35K-03; β-endorphin, substance P, and oxytocin). The limit of detection (LOD) differed for each analyte and ranged from 0.17 to 20 pg/ml for the analytes used in our study. Majority of our data points (96%) fell within the standard curve for the specified analyte. Any sample falling above the standard curve was removed from the analysis. Samples with censored values (below LOD), comprising <1% of our dataset, were reported as lowest standard value divided by 2.

### ELISA assay for the detection of maternal HCV IgG and IgM antibodies

HCV immunoglobulin G (IgG) and immunoglobulin M (IgM) antibodies were quantified in maternal plasma collected at the time of admission for delivery. We performed an indirect enzyme-linked immunosorbent assay (ELISA). Clear 96-well, high-binding polystyrene ELISA plates were coated with 1 ug/ml HCV combined recombinant antigen (cat. no. 228-10628; RayBiotech, Peachtree Corners, GA, USA) in phosphate-buffered saline (PBS) overnight at 4°C. The plates were brought to room temperature and the unbound antigen removed by flicking and blocked by adding 200 μl/well of blocking buffer for 1 h at room temperature. Maternal plasma was heat-inactivated for 30 min and added to duplicate wells in blocking buffer at ratios of 1:100 and 1:50 for IgG and IgM detection, respectively. The responses were visualized by adding horseradish peroxidase (HRP)–anti-human IgG or IgM (BD Pharmingen, San Diego, CA, USA). The reaction was visualized using *o*-phenylenediamine (OPD)/30% H_2_O_2_. The reaction was stopped after incubation at room temperature for 20 min with the addition of 1 M HCl. The optical density (OD) was read at 490 nm absorbance on the SpectroMax^®^ iD3 plate reader (Molecular Devices, San Jose, CA, USA). Positive and negative plasma controls were used to determine suitable cutoff values for IgG and IgM (HCV IgG: OD > 0.096; HCV IgM: OD > 0.408).

### Maternal HCV viral load detection by RT-PCR

To evaluate active acute infection, the HCV viral load was assessed in maternal plasma collected at the time of admission for delivery. We first extracted RNA from samples with positive HCV IgG (OD > 0.096) or HCV IgM (OD > 0.408) determined by the indirect ELISA outlined above. RNA extraction was performed using the Quick-RNA™ Viral 96 Kit (cat. no. R1041; Zymo Research, Irvine, CA, USA) and reverse-transcribed (RT) using the High-Capacity cDNA Reverse Transcription Kit (cat. no. 4368814; Applied Biosystems, Carlsbad, CA, USA) following the manufacturer’s instructions and ran on the Nexus Gradient MasterCycler (Eppendorf, Hamburg, Germany). The RT cycling conditions were as follows: 10 min at 25°C, 120 min at 37°C, and 5 min at 85°C, followed by a 4°C hold. The resulting viral cDNA was then amplified with HCV 5′-UTR/partial core specific forward and reverse primers—HCV_5UTR_1_F: 5′-GTC TAG CCA TGG CGT TAG TAT GAG TG-3′ [melting temperature (TM) = 58.8°C, molecular weight (MW) = 8,057.3 g mol^−1^] and HCV_5UTR_1_R: 5′-ACA AGT AAA CTC CAC CAA CGA G-3′ (TM = 57.0°C, MW = 7,588 g mol^−1^)—using the Platinum™ SuperFi II PCR Master Mix (cat. no. 12368010; Invitrogen, Carlsbad, CA, USA). cDNA amplification was performed as follows: initial denaturation at 98°C for 10 s; 35 cycles of denaturation at 98°C for 10 s, annealing at 60°C for 10 s, and extension at 72°C for 30 s; and a final extension at 72°C for 5 min. The products were run on a 1.5% agarose gel alongside the extraction, RT-PCR, and cDNA PCR controls in triplicate and imaged with GelRed^®^ Nucleic Acid Stain (cat. no. 41003; Biotium, Fremont, CA, USA) using the Azure 300 Imager (Azure Biosystems, Dublin, CA, USA). Samples were considered positive for HCV viral RNA if an amplification product corresponding to 374 bp was detected.

### Phenotyping by flow cytometry

Thawed UCBMCs with >70% viability were thoroughly washed with FACS buffer and stained with Ghost UV450 viability dye (1:100; Cytek Biosciences, Fremont, CA, USA) for 30 min at 4°C. Cells were washed twice with FACS buffer and stained with the following surface antibodies in 50 μl/sample of Brilliant Stain Buffer (1:20; BD Biosciences, Franklin Lakes, NJ, USA): CD3 (BV650), CD4 (CFluorV610), CD8 (CFB548), CD45RA (BV570), CCR7 (BV785), CD20 (PerCPCy5.5), CD27 (CFYG584), IgD (BV605), CD56 (BUV 615), HLA-DR (APC-Cy7), CD14 (BV711), CD16 (BUV395), CD123 (PE-CF594), and CD11c (BUV661), as well as True-Stain Monocytes Block and Human TruStainFc (1:20; BioLegend, San Diego, CA, USA). After 30 min incubation at 4°C, UCBMCs were fixed for 2 h (Tonbo fix/permeabilization solution, 1:3), permeabilized (Tonbo permeabilization buffer), and stained with ki-67 (BV421) intranuclear antibody (1:20) overnight at 4°C. Stained UCBMCs were washed with FACS buffer and ran on the Cytek Aurora flow cytometer (Cytek Biosciences, Fremont, CA, USA) with five lasers using the SpectroFlo software v2.2.0.2. The extracted FCS files were analyzed using FlowJo 10.10.0.

### *Ex vivo* stimulation and intracellular cytokine staining

To measure the myeloid cell responses, 1 × 10^6^ thawed UCBMCs were stimulated for 16 h at 37°C in 10% FBS/RPMI in the presence or absence of bacterial Toll-like receptor (TLR) cocktail (1 mg/ml LPS, 2 mg/ml Pam3CSK4, and 1 mg/ml FSL-1) or viral TLR cocktail (50 μg/ml ssRNA, 5 mg/ml imiquimod, and 1 mg/ml ODN2216). Brefeldin A was added after 1 h incubation and cultured for an additional 15 h at 37°C. Leukocytes were stained with the following surface antibodies for 30 min at 4°C: CD3, CD20, HLA-DR, CD14, CD56, CD16, CD11c, and CD123 (1:20; BioLegend). Samples were fixed and permeabilized using fixation and permeabilization wash buffer (BioLegend) at 4°C for 20 min and stained intracellularly for 4 h: TNFα, IL-6, IL-1β, and MIP1β (CCL4) (1:20; BioLegend). For natural killer (NK)/T-cell responses, 1 × 10^6^ thawed UCBMCs were stimulated for 6 h at 37°C in 10% FBS/RPMI ± phorbol 12-myristate 13-acetate (PMA)/ionomycin. Brefeldin A was added after 1 h incubation and cultured for an additional 15 h at 37°C. Cells were stained with the following surface antibodies at 1:20 for 30 min at 4°C: CD4, CD8, CCR7, CD45RA, HLA-DR, and CD56 (BioLegend). The samples were fixed, permeabilized, and stained intracellularly for IL-2, IL-17, TNFα, and IFNγ (1:20) for 4 h at 4°C. Cells were then washed and acquired using the Attune NxT Flow Cytometer (ThermoFisher Scientific, Waltham, MA, USA) and analyzed using FlowJo 10.10.0.

### 3′ multiplexed single-cell RNA sequencing

Freshly thawed UCBMCs (1–2 × 10^6^ cells) isolated from control (*n* = 4), POE HCV− (*n* = 5), and POE HCV+ (*n* = 4) UCBs were washed in Dulbecco’s PBS (DPBS) with 2% FBS and incubated with Human TruStain FcX (BioLegend) according to the manufacturer’s instructions. Individual TotalSeq Hashtag Oligo Antibodies (BioLegend) were added and incubated following the manufacturer’s instructions. Pellets were washed twice in 2% FBS/DPBS and pooled together. Cell pools were filtered and counted in duplicate to confirm >80% viability. Cell pools were suspended in 2% FBS/DPBS to a final concentration of 1,500 cells/ul. Single-cell suspensions were then immediately loaded on the 10X Genomics Chromium X Controller with a loading target of 30,000 cells. Libraries were generated using the v3.1 chemistry for gene expression and Single Cell 3′ Feature Barcode Library Kit following the manufacturer’s instructions (10X Genomics, Pleasanton, CA, USA). Libraries were sequenced on Illumina NovaSeq 6000 (San Diego, CA, USA) with a sequencing target of 30,000 gene expression reads and 5,000 feature barcoding reads per cell.

### Single-cell RNA-seq data analysis

Raw sequencing reads were aligned and quantified using CellRanger (v6.0.1; 10X Genomics) with the GRCh38 human reference genome. After alignment, initial quality control, normalization, dimensionality reduction, and cell type annotation were performed using Seurat (v5.1.0) as described previously (17) (**Supplementary Table S2**). Differentially expressed genes (DEGs) between groups (control *vs*. POE HCV− and control *vs*. POE HCV+) were determined using Wilcoxon. The Benjamini–Hochberg procedure was employed for false discovery rate (FDR) control, with the gene inclusion criteria requiring expression in at least 25% of the cells in either group (pct.1 > 0.25, pct.2 > 0.25). DEGs were defined as genes with FDR ≤ 0.05 and an absolute log_2_ fold change cutoff of ±0.4. Pathway and functional enrichment analyses were conducted using Metascape (18) (**Supplementary Table S3**).

### Cell–cell interaction analysis

The R CellChat package (14) was employed to infer probabilities of intercellular communication networks between our scRNA-seq clusters. A CellChat object was generated from a Seurat v5 object with the createCellChat function. Data were preprocessed with the function identifyOverExpressedGenes and identifyOverExpressedInteractions, and communication probabilities between clusters were determined with computeCommunProb (truncatedMean, trim = 0.1, interaction.range = 250, contact.range = 100). Communications were filtered (filterCommunication) to a minimum number of 50 cells and the signaling pathway probabilities calculated (computeCommunProbPathway). Data were aggregated for interaction comparisons between groups (control and the POE HCV− and POE HCV+ groups).

### Statistical analysis

Data analysis from Luminex and flow cytometry assays were performed as follows. Normality was assessed using the Shapiro–Wilk test (*α* = 0.05), and outliers were identified via ROUT analysis (*Q* = 0.1%). Group differences were evaluated using one-way analysis of variance (ANOVA) with Tukey’s honestly significant difference (HSD) test. Tukey’s HSD was selected as it provides strong control of the family-wise error rate and is robust to moderate differences in group sample sizes. All possible pairwise group comparisons (control *vs*. POE HCV−, control *vs*. POE HCV+, and POE HCV− *vs*. POE HCV+) were assessed, and adjusted *p*-values were reported. Statistical significance was defined as *p* < 0.05 after adjustment, with results trending toward significance noted at *p* < 0.1. Categorical data were compared using chi-square tests. We report adjusted (*q*-value) to control for the FDR in our scRNA-seq analysis. All statistical analyses were performed using Prism (GraphPad, Boston, MA, USA) and R.

## Results

### Prenatal opioid exposure is linked to adverse neonatal outcomes

UCB was collected from term deliveries of neonates born to mothers with OUD and controls (**Figure 1A**). Given the high prevalence of maternal HCV infection in our OUD group, neonates were stratified by maternal HCV status as determined with ELISA and RT-PCR from maternal plasma collected at the time of admission for delivery (**Figure 1A**; **Supplementary Table S1**). Due to the lack of HCV infections in the OUD-negative pregnant individuals in our cohort, we were not able to include a HCV+OUD− group as a control (19). For this study, we enrolled 38 controls, 34 OUD+HCV−, and 41 OUD+HCV+ mothers (**Supplementary Table S1**). All mothers with OUD met the criteria for diagnosis of severe OUD according to the Diagnostic and Statistical Manual of Mental Disorders, 5th Edition (DSM-5), with nearly half reporting use of multiple opioids at the time of enrollment (**Figure 1B**). In addition, all mothers with OUD were stabilized on medication-assisted therapies (partial or full opioid agonists) at the time of delivery. Newborns with POE and those with (POE HCV+) and without (POE HCV−) HCV co-exposure were significantly smaller in length at birth, while only the POE HCV+ group had significantly less weight at birth compared with the controls (**Figure 1C**). The rates of admission to the neonatal intensive or abstinence care units (NICU/NACU), length of stay (LOS), and requirement for pharmacological treatment of NOWS were all increased in the POE groups, particularly in those with HCV co-exposure (**Figures 1D–F**). Congenital fetal anomalies, including microcephaly, heart block, stenosis, cleft palate, hypospadias, and club/rocker bottom feet, were more common in POE newborns compared with the controls (**Figure 1G**). Other cohort characteristics are shown in **Supplementary Table S1**. Overall, our findings are consistent with previous reports that POE±HCV is associated with smaller newborns, longer hospitalizations, and higher rates of birth anomalies (20, 21).

**Figure 1:**
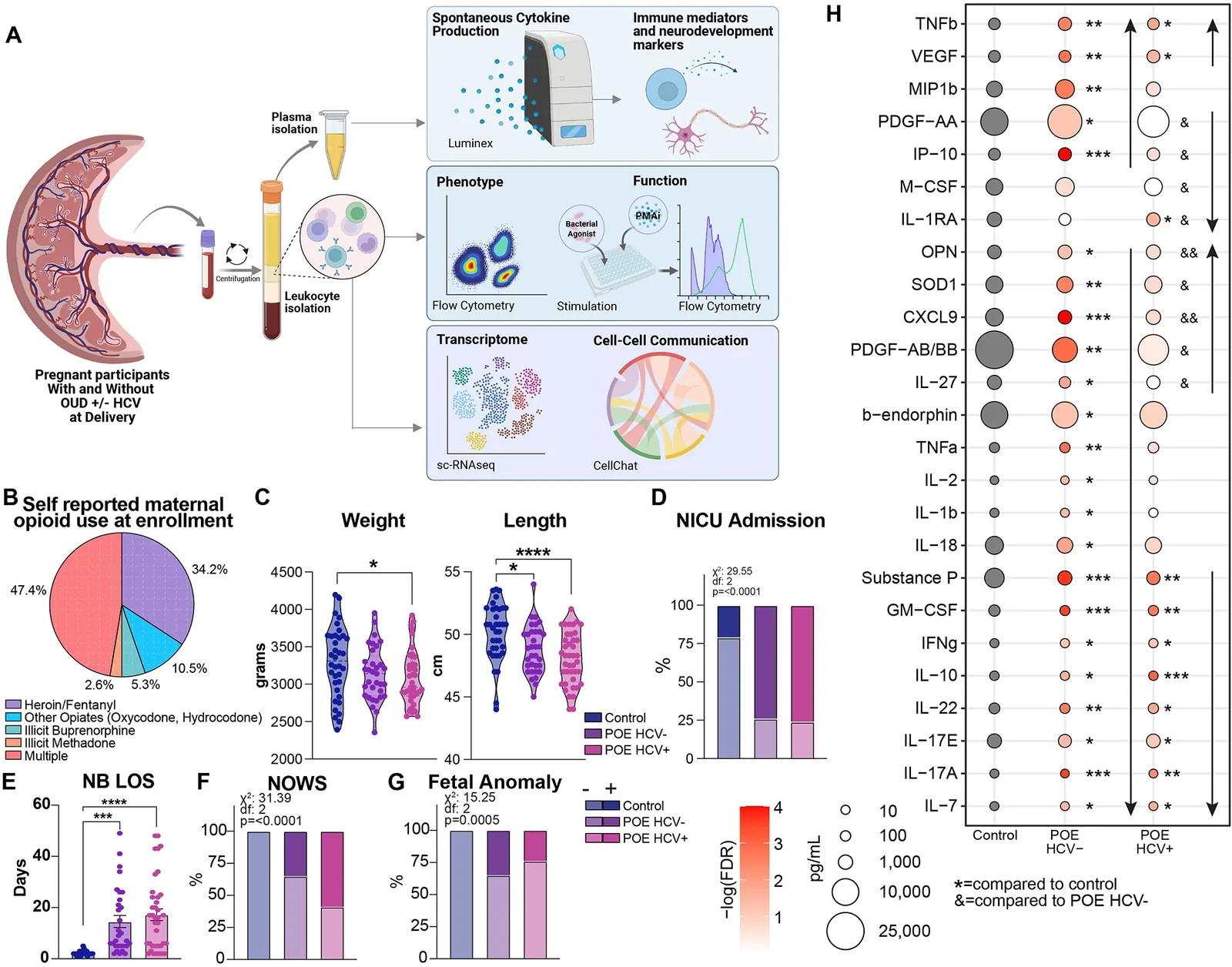
Neonatal outcomes following prenatal exposure to maternal opioid use with and without maternal hepatitis C virus (HCV) infection. **(A)** Experimental design. Umbilical cord blood (UCB) samples were collected from newborns of pregnant participants admitted for labor and delivery. Plasma isolated from UCB samples was assayed for spontaneous cytokine production and neurodevelopmental markers. Isolated leukocytes were subjected to a series of assays to uncover the phenotypic, functional, and transcriptional landscape of UCB immune cells. Immune cell communication networks were determined using CellChat. Findings were stratified by maternal HCV status. **(B)** Pie chart depicting self-reported opioid use at the time of enrollment in the past 1 year (*n* = 75). “*Multiple*” indicates the use of two or more of the listed opioid receptor agonists. **(C)** Violin plots of newborn birth weight (in grams) and length at birth (in centimeters). **(D)** Stacked bar plot of the percentage of newborns admitted to intensive care units (*darker color*). **(E)** Bar plot of newborn length of stay (LOS) in neonatal intensive/abstinence care unit (in days). **(F, G)** Stacked bar plots of the percentage of newborns diagnosed with neonatal opioid withdrawal syndrome (NOWS) **(F)** or fetal anomalies (congenital defects; *darker color*) **(G)**. For **(C–G)** *n* = 38 (control), *n* = 34 (POE HCV−), and *n* = 41 (POE HCV+). **(H)** Bubble plot of the concentrations of the immune mediators and neurodevelopmental markers detected in UCB plasma in the control, HCV−, and HCV+ samples. The *color* represents significance [−log(*p*-value)], while the *size of the bubble* represents the average concentration (in picograms per milliliter) of the shown analyte. *Asterisks* indicate significant differences in the POE HCV−/HCV+ concentrations compared with the controls. *Ampersands* denote significant differences in the POE HCV+ concentrations compared with POE HCV−. Adjusted *p*-values: *^/&^*p* < 0.05; **^/&&^*p* < 0.01; ***^/&&&^*p* < 0.001; ****^/&&&&^*p* < 0.0001. *Arrows* indicate the direction of the significant difference: an *upward arrow* signifies a significantly higher concentration, while a *downward arrow* denotes a significantly lower concentration for the indicated comparison. Quantitative data were examined for normal distribution (Shapiro–Wilk), outliers (ROUT, *Q* = 0.1%), before one-way ANOVA with Tukey’s correction. Categorical data (chi-square) comparisons were performed as described in “*Materials and methods*.”.

### POE ± HCV alters neonatal immune mediators and neurodevelopmental markers

The levels of immune mediators in newborn plasma have been used as valuable predictors of susceptibility to infections and allergic diseases later in life (22, 23). Multiplex analysis of the immune mediators detected in UCB plasma demonstrated POE- and HCV-associated changes. Across the POE groups, the levels of TNFβ and VEGF were elevated, suggesting heightened immune regulation or responses to inflammation/infection. In addition, both POE groups demonstrated decreases in plasma cytokine levels (IFNγ, IL-10, and IL-17A) and growth factor GM-CSF (**Figure 1H**; **Supplementary Figure S1B**). POE without HCV exposure was further characterized by elevated pro-inflammatory factors, MIP-1β, PDGF-AA, and IP-10 with a decrease in TNFα, IL-1β, and IL-18. In contrast, POE HCV+ showed reduced levels of IL-1RA compared with the controls (**Figure 1H**; **Supplementary Figure S1B**).

Furthermore, newborns with POE are at an increased risk of neurodevelopmental delays (24–27), and markers of neurological function can be detected in neonatal plasma (28, 29). Therefore, we measured the levels of the factors important for promoting neurogenesis, mediating neuroprotection, and adaptation to hypoxia (**Figure 1H**; **Supplementary Figure S1B**). Regardless of the maternal HCV status, UCB plasma with POE had reduced levels of substance P. The level of β-endorphin was decreased in the POE+HCV− group compared with the controls. Interestingly, the levels of SOD1 and OPN had differing dynamics with maternal HCV status, with decreased levels without concurrent infection and increased levels with maternal HCV infection (**Figure 1H**; **Supplementary Figure S1B**).

### POE ± HCV differentially alters neonatal immune cell frequency and activation

Analysis of the UCB immune cell phenotype and functional responses using flow cytometry (**Supplementary Figure S1A**) showed substantial remodeling of the adaptive immunity in POE newborns (**Figures 2A–H**). The CD4^+^ T-cell frequencies were significantly increased in POE HCV+ neonates (**Figure 2A**), with a marked shift toward central memory (CM) subsets (**Figure 2B**). Separately, POE HCV− CD4^+^ T cells were hyperresponsive to stimulation relative to POE HCV+ (**Figure 2C**). Similarly, the CD8^+^ T-cell frequencies were increased in both POE subgroups (**Figure 2D**), enriched for CM and effector memory (EM) cells (**Figure 2E**), and hyperreactive to stimulation in POE HCV− newborns (**Figure 2F**). Finally, B cells were significantly expanded in POE HCV+ neonates, driven by replacement of naive subsets by memory B cells (**Figures 2G, H**). The expanded immune memory subsets at birth are indicative of aberrant proliferation and differentiation likely mediated by a heightened maternal inflammatory state. Increased frequency of memory lymphocytes could also shape long-term infection risks or immune-mediated diseases by altering early immune programming (30).

**Figure 2:**
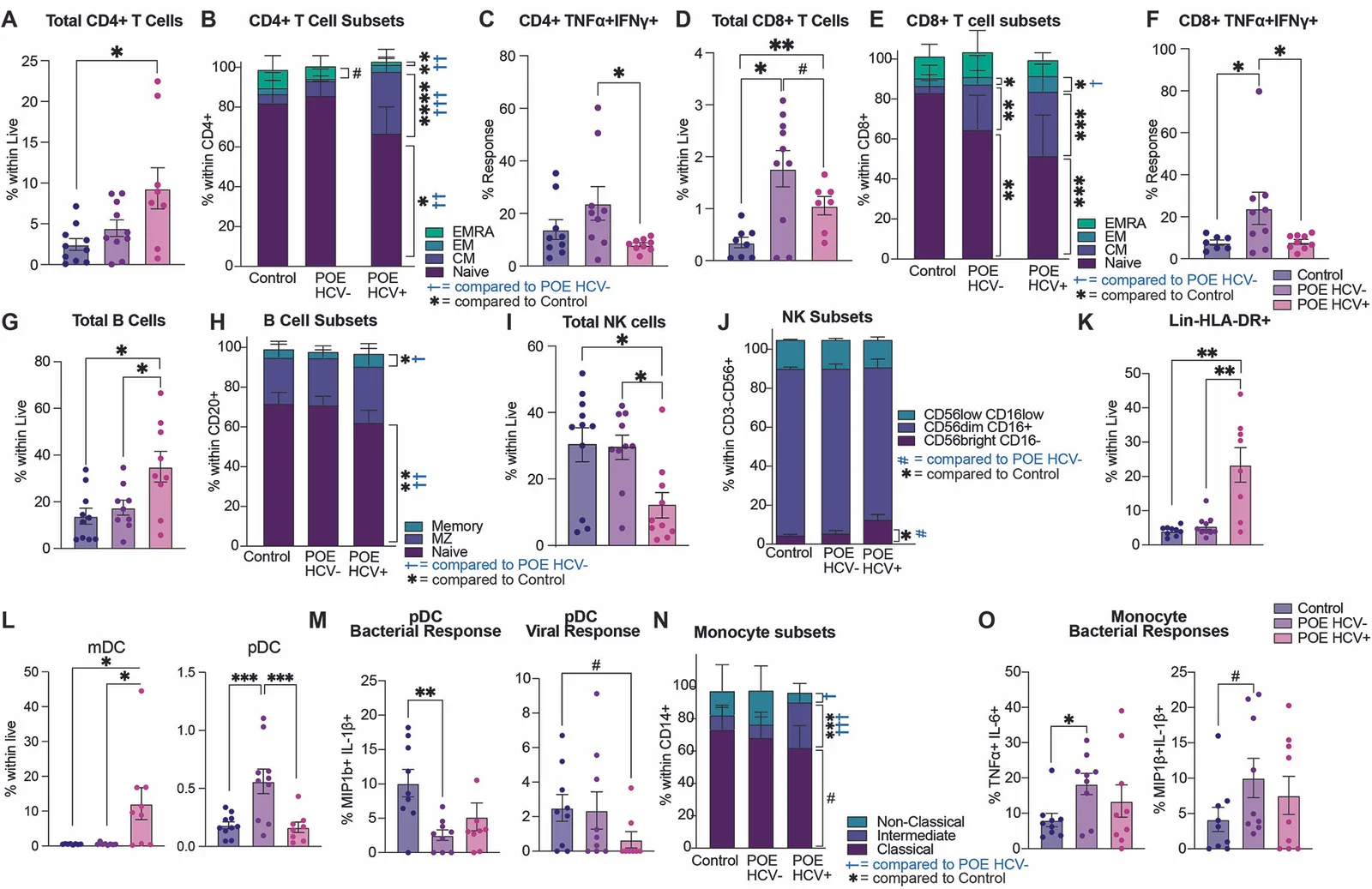
POE±HCV (prenatal opioid exposure with and without hepatitis C virus infection) alters umbilical cord blood (UCB) immune cell frequency and function. **(A)** Bar plot of the percentage of total CD4^+^ T cells within total live cells. **(B)** Stacked bar plot of the percentage of the CD4^+^ T-cell memory subsets (*EMRA*, terminally differentiated effector memory; *EM*, effector memory; *CM*, central memory) within total CD4^+^ cells. **(C)** Bar plot of the percentage of TNFα+IFNγ+ CD4^+^ cells in response to stimulation with the CD3/CD28 activator. **(D)** Bar plot of the percentage of total CD8^+^ T cells within total live cells. **(E)** Stacked bar plot of the percentage of CD8^+^ T-cell memory subsets (EMRA, EM, and CM) within total CD8^+^ cells. **(F)** Bar plot of the percentage of TNFα+IFNγ+ CD8^+^ T cells in response to stimulation. **(G)** Bar plot of the percentage of total B cells within total live cells. **(H)** Stacked bar plot of the percentage of the B-cell memory subsets (*MZ*, marginal zone) within total CD20^+^ cells. **(I)** Bar plot of the percentage of total natural killer (NK) cells (CD3^−^CD56^+^) within live cells. **(J)** Stacked bar plot of the percentage of the NK cell subsets within total CD3^−^CD56^+^ cells. **(K)** Bar plot of the percentage of Lin^−^HLA-DR^+^ cells within total live cells. **(L)** Bar plot of the percentage of myeloid dendritic cells (mDC; *left*) and plasmacytoid dendritic cells (pDC; *right*) within total live cells. **(M)** Bar plot of the percentage of MIP1β+IL-1β+ pDCs in response to stimulation with bacterial (*left*) or viral (*right*) ligands. **(N)** Stacked bar plot of the percentage of monocyte cell subsets within total CD14^+^ cells. **(O)** Bar plot of the percentage of TNFα+IL-6+ (*left*) and MIP1β+IL-1β+ (*right*) monocytes in response to stimulation with bacterial ligands. For phenotyping and *ex vivo* stimulation assays: *n* = 10 (control), *n* = 10 (POE HCV−), and *n* = 9 (POE HCV+). ^#^*p* < 0.1; *^/†^*p* < 0.05; **^/††^*p* < 0.01; ***^/†††^*p* < 0.001; ****^/††††^*p* < 0.0001. Quantitative data were examined for normal distribution (Shapiro–Wilk), outliers (ROUT, *Q* = 0.1%), before one-way ANOVA with Tukey’s correction.

The innate immune compartment also displayed marked changes in phenotype and function with POE±HCV (**Figures 2I–O**). The abundance of NK (CD3^−^CD56^+^) cells was reduced in POE HCV+ UCB, with relative expansion of the cytokine-producing CD56^bright^ subsets (**Figures 2I, J**). The frequencies of Lin^−^HLA-DR^+^ cells, likely dendritic cells (DCs), increased in POE±HCV neonates; however, this was driven by different subsets, with myeloid DCs (mDCs) expanding in the POE HCV+ group and plasmacytoid DCs (pDCs) expanding in the POE HCV− group at the expense of mDCs (**Figures 2K, L**; **Supplementary Figure S1C**). Both DC subsets showed diminished responsiveness to viral TLR ligands, suggesting impaired host defense (**Figure 2M**; **Supplementary Figure S1D**). POE HCV+ had marginally reduced overall monocyte levels (**Supplementary Figure S1E**), with expansion of the intermediate subsets and a decline in the classical and non-classical subsets (**Figure 2N**). While monocytes from POE HCV− neonates displayed heightened responsiveness to bacterial stimulation (**Figure 2O**), those from POE HCV+ neonates were hyporesponsive to viral stimulation (**Supplementary Figure S1F**), indicating that maternal HCV may blunt the antiviral capability despite promoting inflammation. Collectively, these findings suggest that POE alone is associated with hyper-inflammatory innate activation, while HCV co-exposure is associated with depleted effector functions.

### POE ± HCV exposure rewires the transcriptional landscape of UCB adaptive immune cells towards enhanced inflammatory and antiviral processes

To further investigate the relationship between POE±HCV and alterations in the UCB immune cell populations, we performed scRNA-seq on total UCBMCs. A total of 14 distinct immune cell clusters were identified from the transcriptomic data (**Figures 3A, B**). The abundance of immune cell clusters shifted with POE±HCV, notably an expansion of naive B cells with POE HCV− and classical monocytes with POE HCV+ compared with the control (**Figure 3C**). Analysis of the DEGs between each POE group and controls and subsequent enrichment to Gene Ontology (GO) terms demonstrated unique and shared transcriptional signatures among subsets, as represented by Venn diagrams. Across adaptive immune cells, the highest number of DEGs were consistently shown to be between the POE HCV− and control groups (**Figures 3D–F**; **Supplementary Figures S2A, B**).

**Figure 3:**
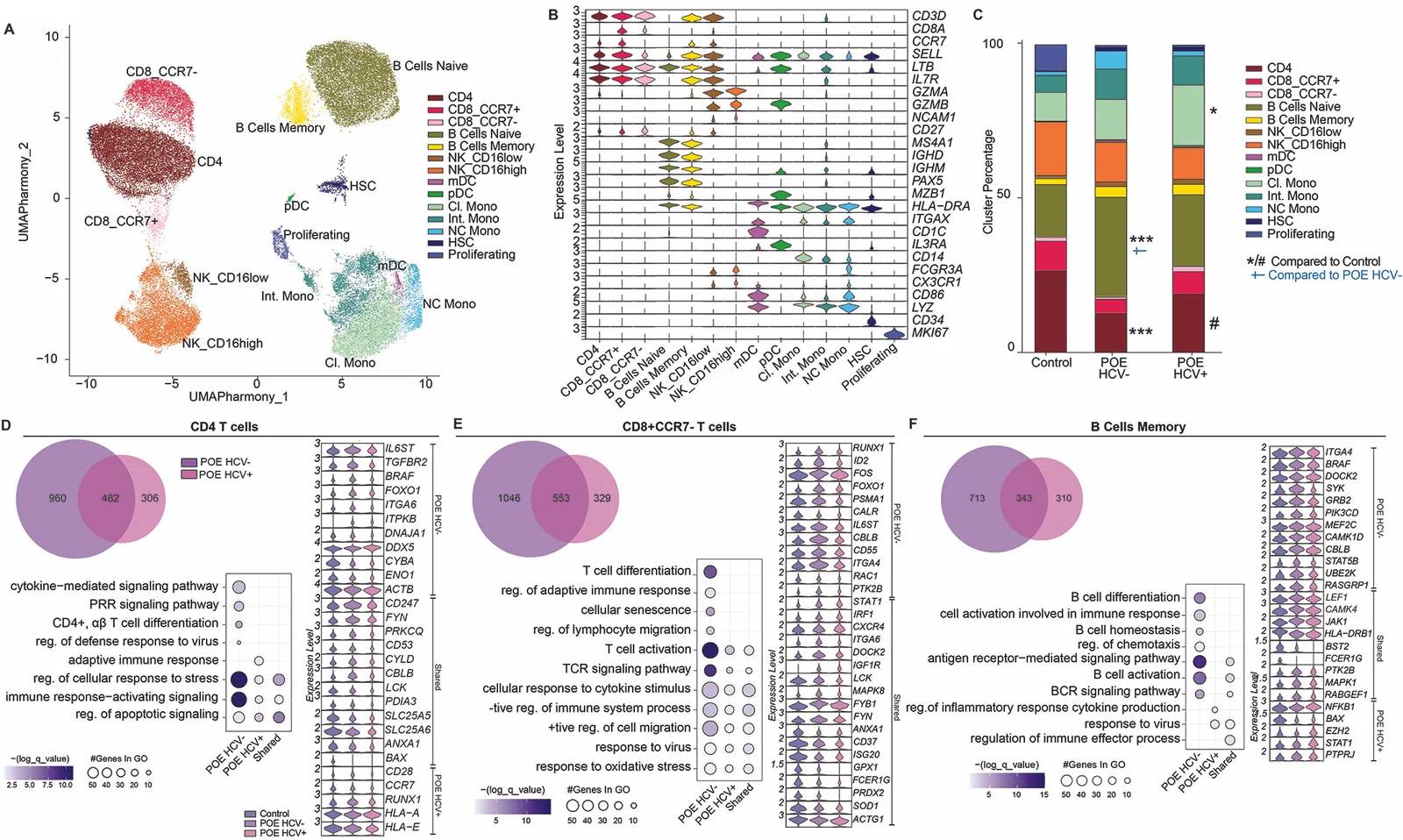
POE±HCV (prenatal opioid exposure with and without hepatitis C virus infection) rewires the transcriptional profiles of umbilical cord blood (UCB) adaptive immune cells. **(A)** UMAP projection of the cell subsets within UCB leukocytes (47,704 cells). Control, *n* = 4; POE HCV−, *n* = 5; POE HCV+, *n* = 4. **(B)** Violin plots showing the expression levels of the marker genes used for cluster identification. **(C)** Stacked bar graph of the UCB leukocyte cluster frequencies (control, 6,943 cells; POE HCV−, 20,162 cells; POE HCV+, 20,599 cells). For *p*-values, *black* symbols represent comparisons to the control, *while blue* denotes comparison between POE groups, with symbols indicating the following: ^#^*p* < 0.1; *^/†^*p* < 0.05; ***p < 0.001. **(D–F)** *Top*: Venn diagram depicting the number of differentially expressed genes (DEGs) for the shown comparisons: POE HCV− *vs*. control (*purple*), POE HCV+ *vs*. control (*pink*), and shared between POE HCV− and POE HCV+ *vs*. control (*overlapping*). *Bottom*: bubble plots of select Gene Ontology (GO) terms from the enrichment of the DEGs. *Right*: violin plots depicting the expression level of representative DEGs for CD4 T cells **(D)**, CD8^+^CCR7^−^ T cells **(E)**, and B cell memory clusters **(F)**. For the bubble plots, the *size of the bubble* denotes the number of genes, whereas the *color* represents significance [log10(*q*-value)].

Within the CD4 T-cell subset, the upregulated DEGs mapped to immune activation (*CD247*, *FYN*, *PRKCQ*, and *CD53*) and apoptosis (*CLYD*, *CBLB*, and *LCK*), whereas the downregulated DEGs mapped to regulation of cellular stress response (*PDIA3*, *SLC25A5/6*, *ANXA1*, and *BAX*) in both POE groups relative to the control (**Figure 3D**). The upregulated DEGs unique to the POE HCV− group mapped to cytokine-mediated signaling (*IL6ST*, *TGFBR2*, and *BRAF*), αβ T-cell differentiation involved in immune response (*FOXO1* and *ITGA6*), and regulation of defense response to virus (*ITPKB*, *DNAJA1*, and *DDX5*), whereas genes associated with pattern recognition receptor signaling (*CYBA*, *ENO1*, and *ACTB*) were downregulated (**Figure 3D**). DEGs unique to the POE HCV+ group included those involved in adaptive immune responses, with an upregulation of *CD28*, *CCR7*, and *RUNX1*, but a downregulation of HLA molecules and *CD27* (**Figure 3D**). Overall, the CD4 T cells in both POE groups showed increased expression of the genes linked to T-cell activation signaling but impaired stress response pathways, while POE alone was associated with heightened viral defenses and cytokine signaling.

Among the two CD8 T-cell subsets, the DEGs in the CD8^+^CCR7^−^ T-cell cluster enriched to multiple shared immune processes between POE groups (**Figure 3E**). For example, genes associated with cellular response to cytokine stimulus (*STAT1* and *IRF1*), positive regulation of cell migration (*CXCR4*, *ITGA6*, and *DOCK2*), and T-cell proliferation, activation, and signaling (*IGF1R*, *LCK*, *MAPK8*, *FYB1*, and *FYN*) were all upregulated in both POE groups relative to the control (**Figure 3E**). On the other hand, genes associated with negative regulation of immune system processes (*ANXA1* and *CD37*) and response to viruses (*ISG20*, *GPX1*, and *FCER1G*) and oxidative stress (*PRDX2*, *SOD1*, and *ACTG1*) were downregulated in both POE groups relative to the control (**Figure 3E**). Upregulated DEGs that were unique in the POE HCV− group compared with the control mapped to T-cell differentiation (*RUNX1, ID2*, and *FOS*), cellular senescence (*FOXO1, PSMA1*, and *CALR*), and regulation of adaptive immune response (*IL6ST, CBLB*, and *CD55*) and lymphocyte migration (*ITGA4, RAC1*, and *PTK2B*). No notable DEGs were identified in the POE HCV+ group relative to the control (**Figure 3E**). Altogether, POE was associated with heightened activation and proliferation, but dampened viral/stress responses, in CD8^+^CCR7^−^ T cells, while POE in the absence of HCV co-exposure was further associated with cellular senescence and migration.

Subsequently, the upregulated DEGs in the naive B-cell cluster in both POE groups mapped to responses to cytokine stimulus (*STAT1, IFI16*, and *PARP1*) and to B-cell differentiation (*RUNX1, BLNK*, and *BRAF*) and signaling (*PLCG2, GRB2*, and *VAV3*), but with the downregulated DEGs mapping to exogenous antigen processing/presentation (*HLA* family genes), B-cell activation (*CD40, CD37*, and *CD81*), and apoptotic signaling (*CASP4* and *CALHM6*) (**Supplementary Figure S2A**). The upregulated DEGs specific to the POE HCV− group mapped to defense response to virus (*TRIM22*) and positive regulation of antigen receptor-mediated signaling (*CBLB, USP15*, and *ZFP36L1*), while the downregulated DEGs mapped to oxidative stress responses (*ROMO1* and *BAX*) compared with the control. The upregulated DEGs unique to the POE HCV+ group mapped to positive regulation of immunoglobulin production (*CD22, IKZF3*, and *PAX5*) (**Supplementary Figure S2A**). Overall, these findings show that activation and stress responses are impaired in naive B cells with POE. However, viral responses and antigen-mediated signaling were amplified in POE alone, while HCV co-exposure was further associated with activation signatures such as heightened antibody production.

Finally, the DEGs among the memory B-cell cluster included shared signatures among the POE groups, such as increased expression of genes associated with B-cell activation (*LEF1, CAMK4*, and *JAK1*), effector function (*HLA-DRB1, BST2*, and *FCER1G*), and receptor signaling pathways (*PTK2B, MAPK1*, and *RABGEF1*), compared with the control (**Figure 3F**). The POE HCV− group uniquely showed increased expression of genes associated with cell activation, immune responses (*ITGA4, BRAF*, and *DOCK2*), regulation of chemotaxis (*SYK, GRB2*, and *PIK3CD*), B-cell differentiation (*MEF2C, CAMK1D*, and *CBLB*), and homeostasis (*STAT5B, UBE2K*, and *RASGRP1*) relative to the control (**Figure 3F**). Lastly, the upregulated DEGs unique to the POE HCV+ group mapped to regulation of cytokine production involved in inflammatory responses (*NFKB1, BAX, EZH2, STAT1*, and *PTPRJ*) *versus* the control (**Figure 3F**). Overall, POE with and without HCV co-exposure altered the memory B-cell transcriptional profiles by enhancing the expression of the genes important for B-cell immune activation and inflammatory responses.

### POE ± HCV exposure rewires the transcriptional landscape of UCB innate immune cells

DEGs were identified between the study groups in innate immune cell populations. As described for the lymphocyte cell clusters, the greatest number of DEGs was observed between the POE HCV− group and the controls. An exception was classical monocytes (Cl._Monocytes), where the largest number of DEGs was detected between the POE HCV+ group and the controls (**Figure 4**; **Supplementary Figure S2**). Firstly, similar transcriptional changes were detected within the cytotoxic CD16^high^ NK cell subset (**Figure 4A**). For example, increased expression of genes associated with immune activation (*NFKB1, CD247, PTK2B, CX3CR1, ITGAL*, and *LCK FYN*) and immune effector processes (*DOCK2, ANXA1, RORA, CREBBP*, and *PLCG2*) was observed in both POE groups compared with the control. However, the genes important for pathogen responses, such as NK cell-mediated cytotoxicity (*NKG7, GZMB*, and *CD2*), cell killing (*CASP4, BAX*, and *C1QBP*), and defense response to viruses (*ISG20, BST2*, and *HLA-E*), were downregulated in both POE groups compared with the control (**Figure 4A**). Overall, despite a heightened activation status, POE was associated with a decreased expression of the antimicrobial and defense response genes.

**Figure 4:**
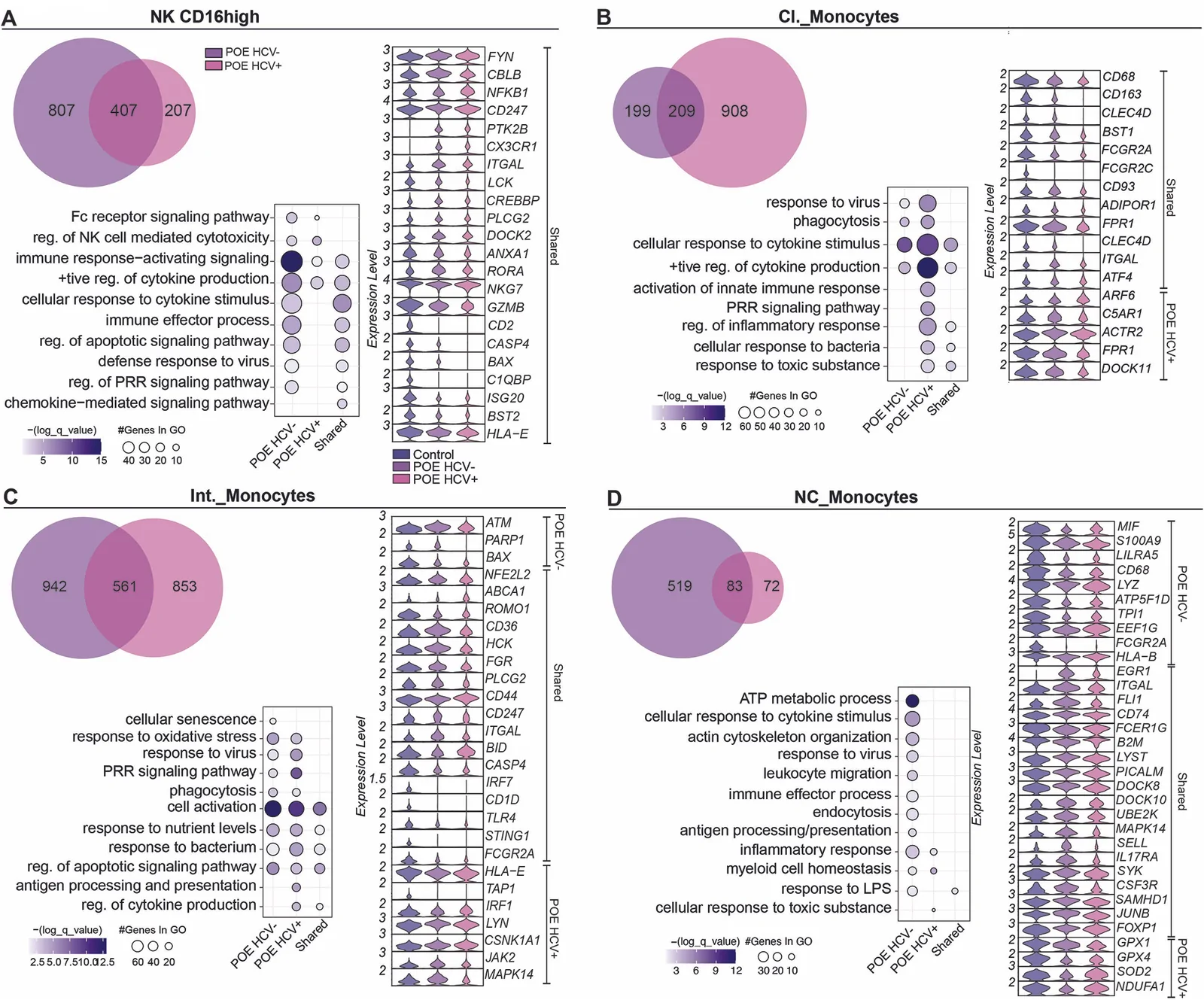
POE±HCV (prenatal opioid exposure with and without hepatitis C virus infection) rewires the transcriptional profiles of umbilical cord blood (UCB) Innate immune cells. **(A–D)** *Top*: Venn diagram depicting the number of differentially expressed genes (DEGs) for the shown comparisons: POE HCV− *vs*. control (*purple*), POE HCV+ *vs*. control (*pink*), and shared between POE HCV− and POE HCV+ *vs*. control (*overlapping*). *Bottom*: bubble plots of select Gene Ontology (GO) terms from the enrichment of the DEGs. *Right*: violin plots depicting the expression level of representative DEGs for the natural killer (NK) CD16^high^ **(A)**, Cl.Monocyte **(B)**, Int._Monocyte **(C)**, and NC._Monocyte **(D)** clusters. For the bubble plots, the *size of the bubble* indicates the number of genes, whereas the *color* represents significance [log10(*q*-value)].

In the cytokine-producing CD16^low^ NK cell population, the upregulated DEGs shared across both POE groups included genes important for leukocyte activation (*RUNX1, FYB1*, and *FYN*), positive regulation of responses to external stimulus (*USP15/34* and *PLCB1*), and cytokine production (*PTK2B* and *CCND3*) relative to the control. However, genes associated with cell killing (*GZMM, NKG7, TYROBP*, and *HLA-C*) were downregulated (**Supplementary Figure S2B**). The upregulated DEGs in the POE HCV− group mapped to cell signaling, including immune response activation (*CD247, PTPRC*, and *DOCK8*), cytokine-mediated (*STAT4, JAK1*, and *TNFAIP3*), and Wnt (*MACF1, ATM*, and *CAMK4*), relative to the control. However, genes mapping to immune effector processes (*C1QBP* and *RPS12*) and regulation of leukocyte migration (*CCL5, SLC25A5*, and *MIF*) were downregulated. Finally, the downregulated DEGs unique to the POE HCV+ group mapped to activation of innate immune responses and regulation of viral processes (*HCST, GAPDH*, and *ISG15*) (**Supplementary Figure S2B**). Overall, both POE groups demonstrated a coordinated shift in CD16^low^ NK cells toward heightened immune activation and signaling, but with decreased expression of the genes key to host defenses relative to the controls.

Among the monocyte subsets, the shared downregulated DEGs in the Cl._Monocyte cluster mapped to phagocytosis (*CD68, CD163*, and *CLEC4D*) and responses to viruses/bacteria (*BST1* and *FCGR2A/C*), cytokine stimulus (*CD93* and *ADIPOR1*), and inflammation (*FPR1* and *CLEC4D*) (**Figure 4B**). However, genes associated with positive regulation of cytokine production (*ITGAL* and *ATF4*) were upregulated in both POE groups relative to the control (**Figure 4B**). No unique DEGs were reported in the POE HCV− group. However, the upregulated genes that were unique to POE HCV+ mapped to activation of innate immune responses (*ARF6, C5AR1*, and *ACTR2*), while genes associated with pattern recognition receptor signaling (*FPR1* and *DOCK11*) were downregulated (**Figure 4B**). Altogether, our findings support that POE, regardless of HCV co-exposure, rewires UCB classical monocytes towards a regulatory state, with HCV exposure further impairing the effector functions and host defenses.

The DEGs in the intermediate monocyte (Int._Monocyte) cluster showed multiple shared signatures among the POE groups (**Figure 4C**). For example, genes important for responses to nutrients and oxidative stress (*NFE2L2, ABCA1*, and *ROMO1*), phagocytosis (*CD36, HCK, FGR*, and *PLCG2*), cell activation (*CD44, CD247*, and *ITGAL*), and apoptotic signaling (*BID* and *CASP4*) were upregulated in both POE groups relative to the control. On the other hand, genes associated with bacterial/viral responses and pattern recognition receptor signaling (*IRF7, CD1D, TLR4, STING*, and *FCGR2A)* were downregulated (**Figure 4C**). In addition, the expression of cellular senescence genes (*ATM, PARP1*, and *BAX*) was increased in the POE HCV− group, while genes associated with antigen processing/presentation (*HLA-E* and *TAP1*) and cytokine production (*IRF1, LYN, CSNK1A1, JAK2*, and *MAPK14*) were all downregulated in the POE HCV+ group relative to the control (**Figure 4C**). Overall, these data suggest that POE alone is associated with disrupted intermediate monocyte function that is further exacerbated by maternal HCV co-exposure.

Lastly, within the non-classical monocyte (NC_monocyte) cluster, the downregulated DEGs mapped to responses to LPS and inflammation (*MIF, S100A9*, and *LILRA5*) and to myeloid cell homeostasis (*CD68* and *LYZ*) in both POE groups relative to the control (**Figure 4D**). Genes mapping to cytoskeletal organization (*EGR1, ITGAL*, and *FLI1*), antigen processing/presentation (*CD74, FCER1G, B2M*, and *LYST*), endocytosis (*PICALM, DOCK8/10*, and *UBE2K*), leukocyte migration (*MAPK14* and *SELL*), and responses to cytokines (*IL17RA, SYK*, and *CSF3R*) and viruses (*SAMHD1, JUNB*, and *FOXP1*) were all uniquely upregulated in POE HCV− (**Figure 4D**). However, genes important for cell metabolism (*ATP5F1D, TPI1*, and *EEF1G*) and immune effector functions (*FCGR2A* and *HLA-B*) were downregulated. Finally, the downregulated DEGs unique to the POE HCV+ group mapped to cellular responses to toxic substances (*GPX1/4, SOD2*, and *NDUFA1*) (**Figure 4D**). These findings suggest that UCB non-classical monocytes are primed towards immune surveillance and pathogen responses with POE alone, while POE and HCV exposure is associated with impaired stress responses.

### Maternal POE impacts UCB immune cell communication networks

CellChat (14) analysis predicted that POE±HCV co-exposure would alter the neonatal immune communication networks (**Figure 5**; **Supplementary Figures S3**, **S4**). While the UCB leukocytes from newborns with POE±HCV displayed an overall increase in the number of ligand–receptor interactions, particularly in the POE HCV− group, the global signaling strength was diminished with POE HCV+ (**Figure 5A**). The largest reductions were observed in the CD8 and NK subsets, particularly with HCV co-exposure (**Figures 5B, C**). In contrast, pDCs became a dominant signaling hub with POE, regardless of maternal HCV status (**Figures 5B, C**). In the controls, the communication networks were enriched for NF-κB signaling (FASLG), inflammatory/stress responses (APOA and GDF), and cell–cell adhesion (TENASCIN and ESAM) (**Figure 5D**; **Supplementary Figure S3A**). However, distinct networks in POE HCV− neonates included those involved in immune cell proliferation and survival (APRIL and IGFBP), cell migration (EPHB and ADGRL), immune tolerance (WNT), and neuroimmune signaling (SLITRK). Furthermore, immunosuppressive protein C/protein C receptor (PROC/PROCR) signaling was unique in POE HCV+ neonates (**Figure 5D**; **Supplementary Figure S3A**). Other noteworthy signaling pathways prominent in both POE groups included those important for cell migration (SEMA6/7, CD6, EPHA, and HSPG), vascular maintenance (ANGPT), intracellular signaling (PTPRM), and OPIOID signaling (**Figure 5D**; **Supplementary Figure S3A**).

**Figure 5:**
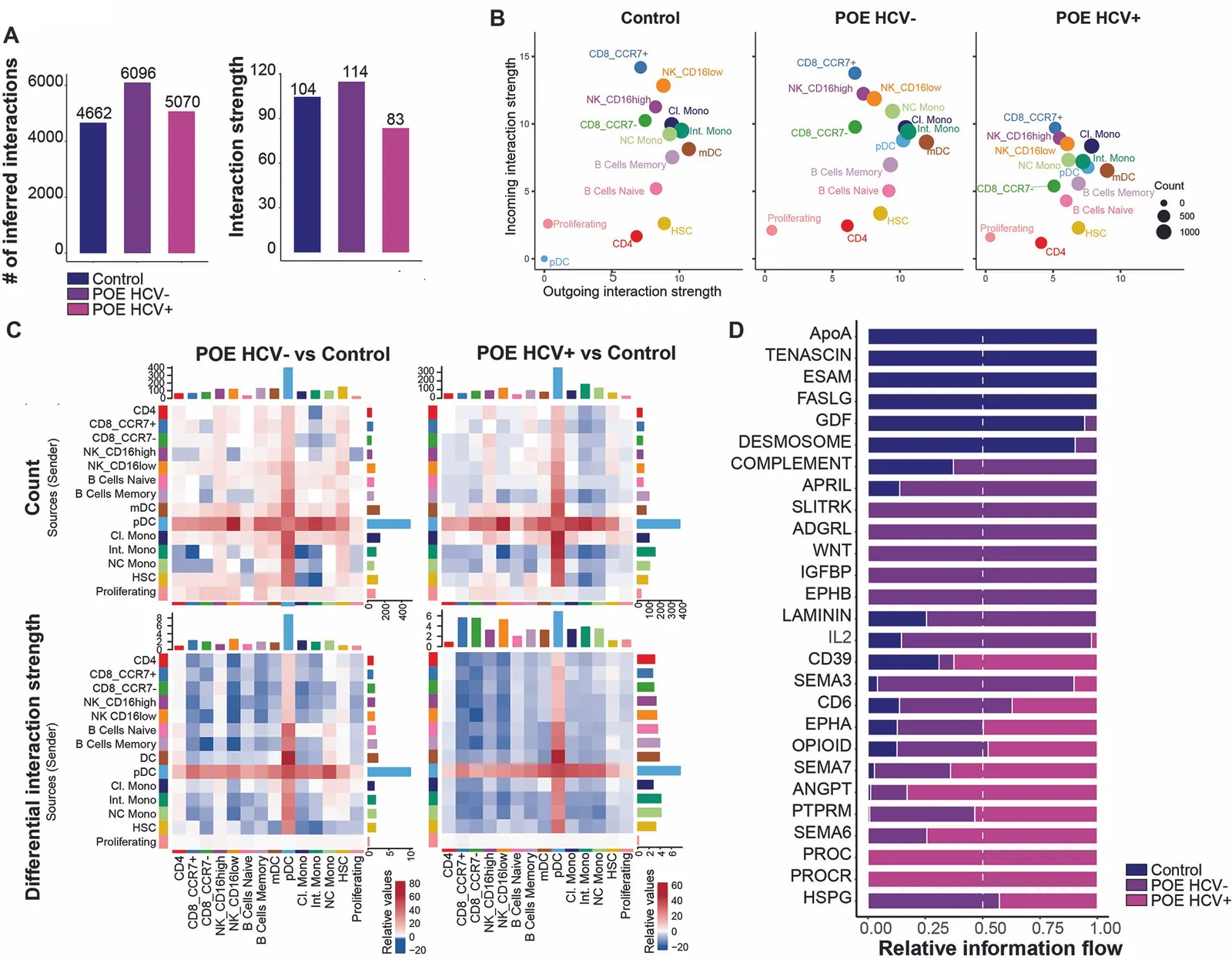
CellChat analysis highlighting the overall signaling patterns between the control and prenatal opioid exposure (POE) groups. **(A)** Bar plot of the number of inferred interactions (*left*) and interaction strength (*right*) between the control (*blue*), POE HCV− (*purple*), and POE HCV+ (*pink*) groups. **(B)** Scatterplot of outgoing (*x*-axis) and incoming (*y*-axis) interaction strength for each annotated immune subset in the control (*left*) and POE (*right*) groups. *Circled area* is proportional to the number of significant ligand–receptor pairs. **(C)** Heatmap of the number of signals (*top*) and differential interaction strength (*bottom*) between the POE HCV− and control (*left*) and POE HCV+ and control (*right*) groups. *Red* and *blue* represent increased and decreased signaling in POE+/− HCV compared with the control, respectively. The bar plots display the sum of the column values (*top bar plot*) or the row values (*right bar plot*) displayed in the heatmap. **(D)** Bar plot of the top signaling pathways ranked by relative information flow (aggregate probability of communication) in the control and POE groups. The *color of the bars* denotes pathways dominant in POE HCV+ (*pink*), POE HCV− (*purple*), and controls (*blue*).

The enhanced pDC signaling in the POE HCV− group was mediated by incoming signals along the APRIL axis from other antigen-presenting cells (**Supplementary Figure S3B**). Interestingly, the pDC subset was also predicted to receive incoming signals from hematopoietic stem cells (HSCs) along the SLITRK axis, thought to play a role in mediating neuroinflammation (**Supplementary Figure S3C**). Outgoing signaling from DCs to HSCs and all other immune cell subsets along the WNT and IGFBP axes, respectively, suggests a regulatory role of DCs in mediating hematopoiesis and immune tolerance with POE (**Supplementary Figures S3D, E**). Unique to the POE HCV+ group, PROCR signaling was predicted to originate from pDCs and was targeted towards memory B cells, CD16^+^ NK cells, and HSCs (**Supplementary Figure S3F**). Interestingly, OPIOID signaling targeted the NK_CD16low subsets in the controls. However, with POE, OPIOID signaling originated mainly from HSCs, DCs, and proliferating cells, distributed broadly across immune populations. Furthermore, the source of DC signaling in the POE groups shifted from mDCs with POE alone to pDCs with POE and HCV exposure (**Supplementary Figure S4A**). We further show the highest expression of the μ-opioid receptor (*OPRM1*) in the controls across the majority of subsets, notably within the NK_CD16low cluster (**Supplementary Figure S4B**). Finally, notable pathways that were shared between both POE groups regardless of maternal HCV status included stronger signaling along the CD6 and ANGPT axes across the majority of cell subsets relative to the control group (**Supplementary Figures S4C, D**).

## Discussion

Maternal environmental exposures, including opioids, can disrupt pregnancy and impair neonatal outcomes, with lifelong consequences (31). Consistent with prior reports (2, 32–34), we found that neonates with POE had lower birth weights, longer ICU stays for treatment of NOWS, and higher rates of fetal anomalies (birth defects). We further demonstrate reductions in the neuroprotective plasma biomarkers—β-endorphin, OPN, SOD1, and substance P—previously linked to neurodevelopmental impairment (27, 35–46). Altered inflammatory mediators were also observed, with reduced levels of growth factors and chemokines with POE and elevated MIP-1β, PDGF-AA, and IP-10 in POE without HCV, which have been linked to an increased risk of inflammatory injury and tissue damage in preterm infants (47, 48). These shifts demonstrate that POE, particularly in the presence of maternal HCV infection, diminishes neuroprotective markers and increases peripheral inflammation. This potentially predisposes neonates to developmental delays and increased vulnerability to infections and inflammatory disorders.

Analyses of the neonatal adaptive immune populations revealed an expansion of memory T cells in both POE groups, possibly due to inflammation-driven homeostatic proliferation *in utero*. This phenomenon of early priming and activation of memory T cells has been reported in cases of maternal immune activation due to infections or immune-related conditions during pregnancy and may have long-term health consequences for offspring (49). However, the CD4 T cells in POE HCV− were hyperresponsive, in contrast to a prior study showing impaired CD4^+^ IL-2 production with POE (13). Transcriptomic data indicated that T cells were skewed toward heightened activation and proliferation, but reduced response to viral ligands and effector capacity, especially with HCV co-exposure. This is consistent with the immune suppression reported in HCV-exposed neonates (50) and potentially underlying long-term immune deficits in newborns with POE and HCV co-exposure (7, 8). Furthermore, in line with our findings of elevated signatures of CD8 T-cell senescence in POE UCB, the expansion of senescent or exhausted CD8 T cells has been described in adults with intravenous drug use, where senescence-associated phenotypes were correlated with an impaired cytokine production and a diminished antiviral capacity (51). In parallel, prolonged opioid exposure has been repeatedly linked to depressed T-cell mitogen responses, altered differentiation, increased apoptosis, and reduced antimicrobial effector functions, indicating that opioids can skew T cells towards a dysfunctional state (52–57). Together with studies showing that prenatal pathogen exposure reshapes the neonatal T-cell compartment and can constrain the CD8 memory pool with poor recall responses (58–60), these findings support a model in which combined opioid and HCV exposure *in utero* imprints dysregulated CD8 T-cell responses that may compromise early life antimicrobial responses and contribute to long-term susceptibility to infection in POE newborns.

Exposure to maternal HCV significantly expanded the UCB memory B-cell populations. This expansion, while potentially beneficial for early pathogen defense, has been associated with increased risks of allergic and autoimmune diseases in later life (61). Despite the increase in the memory populations and transcriptional signatures associated with B-cell activation and inflammatory responses, it is possible that functional responses are hindered akin to immune tolerance, considering that prior studies have reported that maternal HCV infection suppresses neonatal B-cell function by impairing antigen presentation and reducing the capacity for antibody secretion (62). Therefore, we posit that a compromised humoral immunity is a likely mechanism underlying the increased risk of infections in children with POE (7).

We observed a reduced total monocyte abundance, but an expansion of the pro-inflammatory intermediate monocyte subsets in POE HCV+, in line with other reports of reduced monocyte populations in neonates with POE (13). While POE alone increased the monocyte response to bacterial ligands, these responses were blunted with POE HCV+, reflecting paradoxical inflammation with impaired regulation. This discrepancy may be due to the influence of signals received from surrounding immune cells (13). The attenuated responses to bacterial stimulation by the monocytes in the POE HCV+ group add to the growing body of literature showing that maternal HCV infection disrupts monocyte function, promoting pro-inflammatory cytokine production while antigen presentation and tissue repair are impaired (62, 63). These findings were further supported by our scRNA-seq data, which showed that POE, particularly with HCV co-exposure, decreased the monocyte effector functions including responses to pathogens and stress.

DCs in early life have a stunted capacity for antigen presentation and co-stimulation of T cells (64, 65). Here, we report a significant expansion of pDCs in the POE HCV− group and an enhanced signaling along pathways important for immune tolerance and homeostasis (WNT, IGFBP, and APRIL). In addition, the blunted responses to stimuli suggest a functional impairment of pDCs, possibly due to receiving stronger signals associated with immune suppression and tolerance. Human studies have shown that POE is associated with an increased infection risk in neonates, while maternal HCV infection is associated with a reduction in the pDC numbers and functional responses in neonates (50). pDCs are critical for antiviral defense, and their depletion may contribute to an impaired response to viral infections in HCV-exposed neonates. Furthermore, the expansion of DCs with reduced function reflects a broader phenomenon of immune tolerance or exhaustion described in both opioid and chronic viral exposures (50, 62).

The decreased NK cell abundance across both POE groups aligns with prior observations of reduced NK cell numbers in preterm or small-for-gestational age newborns, which is linked to late-onset infection risk (66, 67). The scRNA-seq analysis identified a CD16^low^ NK cell population known for strong cytokine production and a CD16^high^ NK cell population important for cytotoxic functions (68). In POE, regardless of the maternal HCV status, these clusters showed diminished transcriptional programs related to activation and cytotoxicity. Animal and human studies similarly demonstrated that POE suppresses NK cell cytotoxicity and impairs cytokine secretion and migration, effects that are primarily mediated by opioid receptor signaling on NK cells (69, 70). Consistent with this mechanism, our dataset showed that CD16^low^ NK cells, which exhibited the most robust μ-opioid receptor expression in the controls, had a markedly reduced receptor expression in POE and POE+HCV infants, suggesting that chronic *in utero* opioid exposure downregulates this pathway and possibly contributes to the diminished activation and cytotoxic transcriptional programs observed in CD16^low^ NK cells. In addition, cytotoxic CD16^high^ NK cells exhibited transcriptional profiles consistent with reduced migration, cytotoxicity, and inflammation. These findings parallel reports in maternal HCV infection where newborn NK cells displayed an exhausted phenotype with impaired cytotoxic and cytokine responses (71) and newborns and adults with HCV showed reduced NK cell IFN-γ production and cytotoxicity (71, 72).

In summary, this study used multimodal approaches to uncover the impact of POE±HCV on the UCB immune landscape at term. Our findings shed light on the complex interplay between immune cell populations, providing possible mechanisms underlying adverse newborn outcomes with exposures to maternal inflammatory conditions. However, our study had limitations. While we were able to control for maternal HCV infection, we were unable to stratify by other maternal factors without compromising statistical power. For instance, parity was significantly higher in both OUD groups compared with the control. The POE HCV− cohort also had a higher pre-pregnancy BMI. Polysubstance use is prevalent in people who use drugs, including high rates of THC, nicotine, alcohol, and stimulant use. The clinical data obtained from patients with polysubstance use present inherent interpretative challenges. These factors can introduce bias and limit causal inference. As such, future studies leveraging multi-sites will be essential to improve the generalizability and mechanistic understanding of polysubstance-related effects in human systems. Moreover, we acknowledge that while UCB is widely used as a more readily accessible surrogate for neonatal peripheral blood, the two compartments are distinct (73). Further studies are needed to evaluate the impact of POE on postnatal samples. Finally, we acknowledge that the cell numbers for our scRNA-seq analysis were unequal across groups, which may have complicated the interpretation of statistical significance. To mitigate this limitation, we controlled the FDR using the Benjamini–Hochberg procedure and restricted analyses to the genes expressed in at least 25% of the cells in either group being compared. The relative abundance of the immune cell clusters identified by scRNA-seq may not directly correspond to the absolute or proportional cell numbers measured by flow cytometry due to differences in detection sensitivity and potential biases inherent to each method (74). Nevertheless, this study addresses a critical knowledge gap and offers a framework and opportunity for future, larger studies to delineate the complex interplay between prenatal exposure to opioids and/or HCV and neonatal immunity.

## Data Availability

The datasets presented in this study can be found in online repositories. The names of the repository/repositories and accession number(s) can be found below: https://www.ncbi.nlm.nih.gov/, PRJNA1234185 doi: 10.17632/83bh82m9m2.1

## Supplementary material

Supplementary Figure 1 POE ± HCV impacts the frequency of immune cells in UCB at delivery. **(A)** Gating strategy used for adaptive and innate immune cell phenotyping. **(B)** representative barplots of factors from the Luminex assay. p-values: * < 0.05, ** < 0.01, *** < 0.001. **(C)** Bar plot of the % of mDC (left) and pDC (right) from within the Lin-HLA-DR+ cells. **(D)** Bar plot of the % of TNFα+IL-6+ mDCs in response to bacterial (left) or viral (right) stimulation. **(E)** Bar plot of the % of CD14+ monocytes within total live cells. **(F)** % of TNFα+IL6+ monocytes in response to viral stimulation. p-values: #=<0.1,Data was tested for normal distribution (Shapiro-Wilk), outliers (ROUT, Q = 0.1%), before a one-way ANOVA with Tukey correction. For phenotyping and ex vivo stimulation assays; Control: N = 10, POE HCV-: N = 10, POE HCV+: N = 9.

Supplementary Figure 2 Exposure to maternal HCV infection further impacts the transcriptional profile of UCB immune cells. **(A, B)** Top: Venn diagram depicting the number of differentially expressed genes (DEGs) for the shown comparisons: POE HCV- *vs*. control (purple), POE HCV+ *vs*. control (pink), and shared between POE HCV- and POE HCV+ *vs*. control (overlapping). Bottom: bubble plots of select GO terms from enrichment of DEGs and Right: violin plots depicting the expression level of representative DEGs for **(A)** B Cells Naive, and **(B)** NK CD16low clusters. For the bubble plots, the size of the bubble represents the number of genes whereas the color represents the significance (log10(q-value)).

Supplementary Figure 3 CellChat analysis highlighting unique ligand-receptor pathways. **(A)** Heatmap displaying the relative signaling strength of specific pathways across cell clusters for control (left) and POE (right) groups. Color bar plot (top) displays total signaling strength of specific cell clusters for all pathways. Grey bar plot (right) displays total signaling strength of specific pathways for all cell clusters. **(B-E)** Network signaling diagram (or chord plot) of **(B)** APRIL, **(C)** SLITRK, **(D)** WNT, and **(E)** IGFBP signaling pathways among cell subsets that are unique to the POE HCV- group. F) Network signaling diagram of the PROCR signaling pathway among cell subsets that are unique to the POE HCV+ group. Nodes represent cell subsets and edge width is proportional to the CellChat communication probability.

Supplementary Figure 4 CellChat analysis highlighting overall signaling patterns between control and POE groups. **(A)** Network signaling diagram of the OPIOID signaling pathway among cell subsets across control (left), POE HCV- (middle), and POE HCV+ (right) groups. **(B)** Bubble plot of the expression of opioid receptor genes across subsets by group. OPRM1: μ-opioid receptor, OPRL1: λ-opioid receptor, OPRD1: δ-opioid receptor, OPRK1: κ-opioid receptor. The color represents the average expression, and the size of the bubble represents the percent expression. (C-D) Network signaling diagram of the **(C)** CD6 and **(D)** ANGPT signaling pathways among cell subsets across control (left), POE HCV- (middle), and POE HCV+ (right) groups.

Supplementary Table 1 Cohort information.

Supplementary Table 2 Marker gene lists supporting the single-cell RNA sequencing data in **Figure 3**.

Supplementary Table 3 Differentially expressed gene (DEG) lists supporting the single-cell RNA sequencing data in **Figures 3**, **4**, **Supplementary Figure S2**.
